# Healthcare provider experiences interacting with survivors of intimate partner violence: a qualitative study to inform survivor-centered approaches

**DOI:** 10.1186/s12905-023-02700-w

**Published:** 2023-11-08

**Authors:** Ronald Anguzu, Laura D. Cassidy, Annettee O. Nakimuli, Judith Kansiime, Harriet M. Babikako, Kirsten M. M. Beyer, Rebekah J. Walker, Christopher Wandira, Felix Kizito, Julia Dickson-Gomez

**Affiliations:** 1 Division of Epidemiology and Social Sciences, Institute for Health and Equity, Medical College of Wisconsin, Milwaukee, US; 2 Department of Obstetrics and Gynaecology, School of Medicine, College of Health Sciences, Makerere University, Kampala, Uganda; 3 Action for Community Development (ACODEV), Kampala, Uganda; 4 Department of Epidemiology and Biostatistics, Makerere University School of Public Health, Makerere University College of Health Sciences, New Mulago Gate Road, Mulago, Kampala Uganda; 5 Department of Child Health and Development Center, School of Medicine, College of Health Sciences, Makerere University, Hospital Complex, P.O. Box 7072, Mulago Hill, Kampala, Kampala Uganda; 6 Center for Advancing Population Sciences (CAPS), Medical College of Wisconsin, Milwaukee, WI US; 7 Division of General Internal Medicine, Medical College of Wisconsin, Milwaukee, WI US; 8 District Health Office, Luuka District Local Government, Iganga, Uganda

**Keywords:** Intimate partner Violence, Survivor-centered approach, Qualitative study, Uganda

## Abstract

**Background:**

Intimate partner violence (IPV) remains a pervasive form of gender-based violence (GBV) that is largely undisclosed, especially among women seeking healthcare services in Uganda. Prioritizing survivor needs may improve IPV disclosure. This study explores healthcare worker experiences from provider-patient interactions with survivors seeking antenatal care services (ANC) in Uganda.

**Methods:**

In-depth interviews were conducted among twenty-eight experienced healthcare providers in a rural and an urban-based ANC clinic in Eastern and Central Uganda. Providers were asked what they viewed as the needs and fears of women identified as having experienced any form of IPV. Iterative, inductive/deductive thematic analysis was conducted to discover themes regarding perceived needs, fears, and normalizing violence experienced by IPV survivors.

**Results:**

According to healthcare providers, IPV survivors are unaware of available support services, and have need for support services. Providers reported that some survivors were afraid of the consequences of IPV disclosure namely, community stigma, worries about personal and their children’s safety, retaliatory abuse, fear of losing their marriage, and partners’ financial support. Women survivors also blamed themselves for IPV. Contextual factors underlying survivor concerns included the socio-economic environment that ‘normalizes’ violence, namely, some cultural norms condoning violence, and survivors’ unawareness of their human rights due to self-blame and shame for abuse.

**Conclusions:**

We underscore a need to empower IPV survivors by prioritizing their needs. Results highlight opportunities to create a responsive healthcare environment that fosters IPV disclosure while addressing survivors’ immediate medical and psychosocial needs, and safety concerns. Our findings will inform GBV prevention and response strategies that integrate survivor-centered approaches in Uganda.

## Introduction

According to the World Health Organization (WHO), intimate partner violence (IPV) is a pervasive form of gender-based violence (GBV) that remains a major public health problem [1, 2]. IPV refers to any behaviour within an intimate relationship that results in physical, psychological and/or sexual harm [3]. In Uganda, IPV is predominantly male perpetrated with 29.3% of women experiencing psychological, 22.5% physical, and 16.6% sexual forms of IPV while among men, 8% had ever experienced some form of IPV [4]. All forms of IPV are a human rights violation that affects the dignity, physical and mental wellbeing of its survivors. Violence against women and girls (VAWG) in intimate relationships is mostly driven by differential power dynamics of men over women, particularly due to social norms, attitudes, and patriarchal traditions that perpetuate gender inequality [5]. This Ugandan context within which IPV occurs is of public health importance because female IPV survivors experience persistent socio-cultural, economic, and political barriers. In addition, IPV poses major negative impact on pregnancy and birth outcomes being associated with premature births, low birth weight, postpartum depression symptoms, suicidal ideation, and HIV/sexually transmitted infection acquisition [6, 7].

Help seeking behavior or reporting can reduce the adverse effects of IPV. Although formal resources providing support for IPV are scarce in low- and middle-income countries (LMICs) including Uganda, many IPV survivors prefer using informal resources such as family members, and church leaders than seeking help from health facilities, law enforcement or judiciary [8, 9]. Studies show that most IPV survivors initially seek help from or disclose IPV to their family members, followed by health care workers [4, 9–12]. Besides family as a support resource, more female IPV survivors (10.5%) utilize healthcare services for help than those seeking help from local councils (8.5%), police (2%), social services (0.7%), and legal proceedings (0.2%) [13]. Non-disclosure of IPV is more likely to occur among survivors in post-conflict settings, persons living with HIV, persons living with disabilities, adolescents, and pregnant women [14–16]. Survivors tend not to disclose their experiences of partner abuse because it may trigger traumatic memories or sometimes lead to anxiety due to subsequent perpetrator threats. Non-disclosure of IPV is linked to fear of retaliatory abuse from perpetrating partners and their families [17, 18], societal stigma, and health worker distrust [19, 20]. Similarly, healthcare providers perceive IPV survivors to have inadequate awareness of [21], or substantial individual obstacles hampering survivor accessibility to existing GBV-related services [22, 23].

Non-disclosure to health workers, and low care-seeking behavior following IPV may affect effective responses to IPV. On the other hand, under-detection of IPV by healthcare providers may contribute to underreporting in routine surveillance which affects policy planning and prioritization for resource allocation. Conversely, over-reporting or sharing data on IPV among stakeholders should be conducted in a way that does not jeopardize the safety of survivors [24]. This is because survivors often experience safety concerns and anxiety over worsening intimate partner abuse when they report experiencing IPV. Survivors of partner abuse tend to report IPV to healthcare providers, legal authorities, friends, family members or community leaders [4]. Therefore, patient-physician interaction presents a unique opportunity to identify and support IPV survivors as well as discuss prevention and safety plans.

Providers of GBV prevention services should be aware of, and be responsive to specific IPV survivor needs, namely, survivor ‘*centeredness*’ [25]. Survivor-centered approaches mean that survivors guide the interventions in terms of what, and how to go about meeting their needs [26] which can be empowering to individuals experiencing spousal abuse. Empowering effects of survivor-centered approaches on survivors include provider practices that: (i) ensure survivor safety by preventing and mitigating further violence, (ii) protect confidentiality and rights of survivors when they choose to disclose their experiences of IPV, (iii) demonstrate respect for IPV survivor needs, rights preferred choices, and (iv) promote non-discrimination to ensure survivors have access to and receive needed or appropriate medical, psychosocial, and/or legal support [25, 27–29]. Healthcare workers usually apply medical model of practice when addressing IPV [30]. However, it is important for healthcare workers to consider integrating rights-based approaches to clinical practice when working with individuals who have experienced IPV [31]. In addition, financial empowerment enables survivors become more independent and self-supporting in terms of meeting their own personal and family’s needs [32, 33]. However, healthcare workers should have the capacity to identify and respond appropriately to survivor concerns. This can be achieved with appropriate knowledge, attitudes, and supportive environments to respectfully prioritize survivor’s own experiences [34]. However, there is limited literature demonstrating the needs and fears that healthcare seeking IPV survivors reveal to healthcare providers during routine antenatal care (ANC) visits. Provider perspectives of survivors’ concerns, fears, and needs has the potential to identify actionable targets towards IPV prevention and response in Uganda.

It is critical for support services to stimulate disclosure, and reinforce both survivors’ and providers’ awareness about GBV service availability [35, 36]. In order to respond to this knowledge gap, our first step was to investigate these survivor needs from the perspective of clinicians who are frontline workers, and often the first point of contact for IPV survivors during healthcare facility visits. In this study, we explored the experiences of rural- and urban-based health providers in their interactions with IPV survivors attending ANC in Central and Eastern Uganda.

## Methods

### Study setting

In 2020, we interviewed twenty-eight, purposively selected healthcare providers from rural (Luuka district) and urban (Kampala) settings of Uganda. Luuka district is predominantly a sugarcane-growing community as their main commercial agricultural activity [37]. Kisenyi division, an urban setting in Kampala Capital City Authority (KCCA), central Uganda has several areas of industrialized commercial activity and informal settlements [38]. We purposively selected Kiyunga health center (HC)IV and Kisenyi HCIV because they are the main public, healthcare referral facilities in rural, Luuka district and the urban Kisenyi division in Kampala district respectively. HCIV’s are mid-tiered health facilities in the national referral hierarchy that mainly offer basic prevention, promotion and curative services through outpatient and community outreach and comprehensive Emergency Obstetrics and Newborn Care (CEmONC) services [39–41]. The selected facilities have the highest in- and out-patient attendances among HCIVs in the respective study sites.

### Participant recruitment

We approached the district health officer of Luuka district (rural) and the Director of Medical Services in KCCA that supervises Kisenyi Health HCIV (urban) to obtain clearance to conduct this research in the respective study sites. We recruited study participants as follows. First, at the study sites, the data collector initially introduced the study to the ANC in-charges who subsequently provided their duty call rota which formed our sampling frame comprised of weekly duty schedules of health workers in their ANC clinics. Secondly, our inclusion criteria were clinicians serving as midwives, nurses, psychosocial counsellors, general physicians (medical officers) and obstetricians / gynaecologists (OBGY). These healthcare worker cadres were purposively recruited because they provide maternal health services namely, ANC, labor/delivery, and PNC. We excluded providers who were not on duty during the week that data collection were conducted. Thirdly, ANC health workers were approached to meet the data collector either in-person or through phone calls to ascertain their willingness to participate in this study. Fourth, willing participants shared their availability and selected their preferred location where, and time and date when interviews would be conducted. Lastly, participants were informed that their participate in this study was voluntary and information provided during their interviews would not be shared with supervisors. We obtained written informed consent before conducting the interviews.

### Data collection

**Fig. 1:**
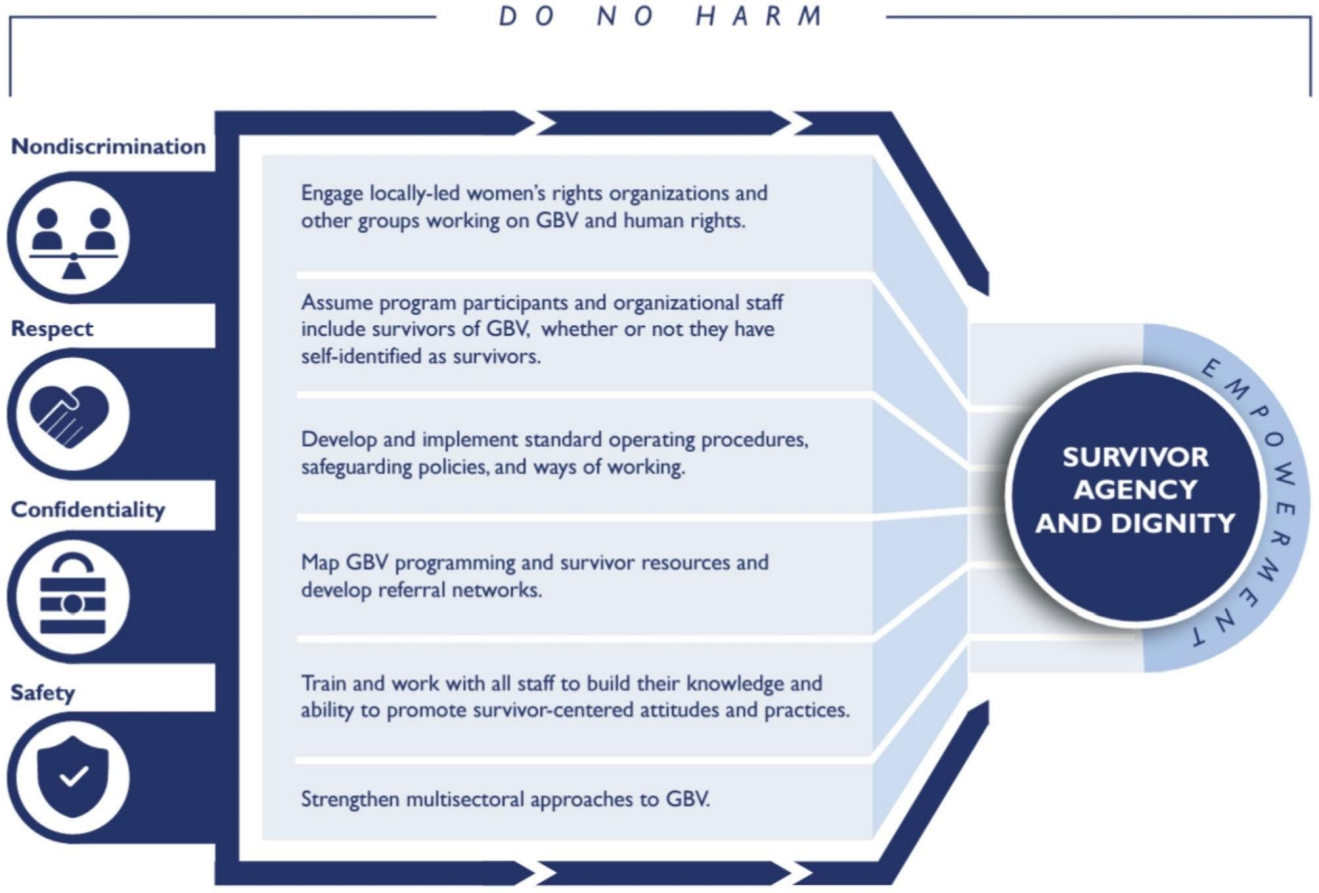
Adopted USAID survivor-centered approach for GBV programming

The duration of interviews ranged between 30 and 90 min and were conducted by the first author trained in conducting qualitative interviews. The interviews were conducted in rooms within ANC clinics that were private and without any interruption. Notes were taken during and immediately after interviews to document non-verbal cues. Participants were given $15 as compensation for their time. Our in-depth interview guide was pre-tested among three respondents in an urban health care facility besides the two health facilities for the current study. We adapted USAID’s GBV survivor-centered framework (Fig. 1) to guide our interview question development. The interview guide contained questions that covered the respondents’ clinical training, clinical practices and experiences that mainly focused on their routine screening for IPV. During the interviews, some respondents made suggestions regarding some questions which were refined during the pre-testing interviews. Probes allowed for flexibility to obtain additional information regarding their clinical practice and experiences regarding routine IPV screening. Probing was also used to explore survivor fears, anxiety, and concerns that IPV survivors disclosed to healthcare providers during clinic visits. Member checking was conducted following inorder to improve validity and representativeness of participants’ responses [42, 43].

### Qualitative data analysis

In-depth interviews were audio-recorded, transcribed verbatim, and analyzed using MAXQDA, a qualitative data analysis software. Inductive-deductive coding of the transcripts was conducted using manifest thematic content analysis by two coders to generate a list of codes. Using iterative processes of reading and re-reading, the final codes were agreed upon. Quotes illustrating the corresponding themes are reported verbatim.

### Ethical considerations

#### Ethical approval

to conduct this study were obtained from The AIDS Support Organization (TASO) IRB, and the Medical College of Wisconsin (MCW) IRB. Administrative clearance to conduct interviews was obtained from the Uganda National Council of Science and Technology (UNCST), Office of the Director of Public and Environmental Health at KCCA, the District Health Office of Luuka district, and in-charges of respective clinics in Kiyunga HCIV and Kisenyi HCIV. Interviews were conducted in a private room to ensure privacy and confidentiality and not disclosed to respondent’s clinic supervisors.

## Results

Twenty-four healthcare providers were interviewed to provide their perspectives of survivor experiences during physician-patient interactions as described in Table 1. Three major themes emerged, namely: (i) perceived survivor needs, (ii) survivor fears/concerns, and (iii) normalizing violence as highlighted in Table 2 and described below.

**Table 1:** Characteristics of healthcare providers in two health facilities, Uganda (N = 28)

Characteristic	Frequency
**Median (IQR) duration of clinical practice, years**	4.0 (0.1–19)
**Gender**	
Male	7
Female	21
**Routine IPV screening**	
No	17
Yes	11
**IPV training**	
No	18
Yes	10
**Clinical specialty**	
Enrolled midwife	14
Registered midwife	8
Medical officer	4
OBGY	2
**Providers’ setting**	
Urban	15
Rural	13

**Table 2:** Emergent themes

Code
Perceived survivor needs
Support services for survivors
Unemployment
Household poverty
Unaware of GBV service availability
Provider’s ability to support survivors
Survivor fears/concerns
Stigma
Fear of disclosure of abuse
Fear of losing their marriage
Fear of losing financial support from partner
Persistent safety concerns
Distrust of healthcare providers
Normalizing violence
Justifying violence
Abuse as expression of affection
Unaware of human rights
Self-blame and shame for abuse

### Perceived survivor needs

#### Support services for survivors

Women who experience IPV tend to express feelings of entrapment by their abusive partners because they may not be able to support themselves or their children financially. These situations highlight the lack of, and the need for medical, psychosocial, and financial support. Our respondents made some suggestions that includes provision of different support services through stakeholder collaboration with non-profit organizations as mentioned in the following quotes.

*What we feel we can provide is the health part of it, but financially I think we cannot. So, we had an organization [non-profit organization] here dealing with the gender-based violence which is in place. When we receive these mothers and we feel they really need help, we refer them to the gender-based violence department which is based at the ART [anti-retroviral therapy] clinic. [Urban, Female, ANC Midwife 9]*

*Maybe to involve other partners like police, if health workers can work hand in hand with the legal team like police, like the LC1s in the place, we are already having VHTs, police, and the political side has a lot of influence from the community like these counselors (local council chairpersons) [Rural, Female, ANC Midwife 7]*.

According to some providers, IPV survivors need psychosocial support to address issues of low self-esteem and helplessness. Respondents expressed optimism about their ability to address psychosocial needs such as self-esteem issues through community awareness raising on GBV. However, one urban-based physician stated that at the healthcare facility level, they lacked the ability to implement this strategy.

*I think the community need sensitization and we need empowering of these women. Some may be submissive. That is where the husband says do not and the woman just accepts that, but we have to empower them [women]. One [psycho-social workers] also need to go to the community, to sensitize the community. We may not be able to do it like for us at our level. [Urban, Male, ANC Nurse 2]*

#### Unemployment

According to our respondents, many IPV survivors are not formally employed. Some of the consequences of unemployment that perpetuates IPV include lack of financial independence, reducing women’s decision-making power in the household and hence making women vulnerable to IPV as stated in the quotes below.

*Most of the woman around here do not work, they depend on the men. However much she is traumatized by the man because of the life she keeps quiet. [Rural, Male, Psychosocial counsellor 15]*

*When you look at the mothers who come for antenatal, 90% have no formal jobs. What does this mean? It means that these mothers cannot financially support themselves. In case there is something with this mother which requires money, that means they only depend on their husbands for financial support. [Rural, Male, General Medical Doctor 3]*

#### Household poverty

Study participants stated that poverty creates stressful environments which results in conflict in their homes and subsequently into violence. This household violence may arise particularly when men face difficulty or are unable fulfil their role as a provider in the family. The excerpts below describe the effects of household poverty that trigger IPV.

*In most cases the root cause to family issues is poverty. So, a woman will say one word because the husband did not buy anything at home and it will spark fire, they will start fighting, but gender-based violence has always been there. [Rural, Male, ANC Nurse 1]*

*Poverty in the society, because if you screen mothers that are coming [to healthcare facilities], most of them who come in with those GBV cases, they are not financially stable. If you try to talk about it with them, they will tell you that it begun when I asked for money for transport, he is no longer taking care of us you know, … most of the GBVs starts like that and That is why people are getting those GBV cases. [Urban, Female, ANC midwife 13*

#### Unaware of GBV service availability

In situations where survivors thought of seeking GBV related support, they often did not know where to get it or where to report abuse experienced while some survivors thought that spousal abuse is not reportable as highlighted below.

*And the other thing is that they don’t know where to go, at the end of the day, if it is not severe, you know it may not be physical but emotional and she doesn’t know where to go. Most people know that you only go to hospital when you have sickness, so they may not come … because they know they will just give me drugs … so there is that lack of knowledge because they do not know where to go. [Rural, Male, General Medical Doctor 4]*

*At times they [survivors] even don’t know where to report. They may not even think of it that if there is violence they have to report. Most of them have that knowledge gap. You have to create awareness down to them and when they get to know they will know where to start from. They have that knowledge gap. [Rural, Female, ANC Midwife 5]*

#### Provider’s ability to support survivors

Respondents revealed that they have training needs that would help them provide or make appropriate referrals to stakeholders for supportive GBV care. This is highlighted in the quote below.

*You have to train us on how we can screen the mothers and where we can refer these mothers, and what can we do with these mothers. It may not be referring but having the skills that we can handle. So, if you can train us in how we can handle when a mother has such a problem, when gender-based violence is at this rate, you can handle like this and this one we cannot handle. [Rural, Female, ANC Midwife 7]*

### Survivor fears/concerns

#### Stigma

According to one provider, since survivors fear being stigmatized following partner abuse, they confide in a few relatives like their parents. Providers felt that they were the least likely person to whom women will disclose.

*When it comes to intimate partner violence, I think there is stigma, and all attached to that. Some people feel offended if you start asking if their partner is violent. So, we leave it to them to bring it forth … Intimate partner violence comes with lots of stigma. When people discuss what has happened to them, they think they will become a laughingstock. So, they prefer not to discuss it. They discuss it with either very close friends or with parents or other relatives but not health workers. What happens in my home stays with me. [Urban, Male, OBGY 6]*

*Some women don’t want to expose their husbands. Sometimes they fear and say that they respect [their intimate partners]. So, when they talk bad words about their husband, when he is there, she will feel ashamed. [Rural, Female, ANC Midwife 7]*

*When you are raped here in Uganda, people will want to sit on it because they will feel very embarrassed coming out to say that I am raped. So, same way this person may not easily come out to tell the doctor that this pregnancy was out of rape. [Urban, Male, OBGY 14]*

### Fear of disclosure of abuse

Several reasons were mentioned as to why some survivors fear to disclose IPV. These emergent sub-themes included survivors fearing to lose their marriage, financial support form their partner, and worries about their safety after disclosure of abuse. According to our respondents, some survivors of school-going age, usually adolescents, fear disclosing rape, retaliatory partner abuse, and whether the perpetrator partners will get imprisoned. This was highlighted below.

*I think they fear to disclose their information … they fear to approach the health workers, if you don’t ask them, some of them fear to talk, but those ones who can talk they can tell you. Those ones who fear even if you ask, they keep Quiet. [Rural, Female, ANC midwife 11*

*They always have a feeling that when they bring the husband to you as a health worker for counselling, it is another way of reporting him to the authorities. In the process, the man will be more annoyed, so when they get back home you will be getting some more torture. [Rural, Male, Psychosocial counsellor 15]*

#### Fear of losing their marriage

Respondents stated that some IPV survivors feared that they would end up divorced or separated. Some of these survivor fears were rooted in distrust of healthcare providers disclosing spousal abuse to the survivors’ partners as described in the quotes below.

*They fear to lose their marriage … Traditionally, cultural beliefs you are not supposed to report your husband when you quarrel and fight you are supposed to keep quiet until now it becomes beyond but, in most cases, people fear to lose their marriage. [Urban, Female, ANC Psychosocial counsellor 16]*

#### Loss of financial support from partner

Respondents mentioned that some IPV survivors feared that they would lose support from their partners, especially financial support, if their spouses were arrested or imprisoned after disclosing IPV to the authorities. Such fears lead some GBV survivors to remain silent and not report or disclose abuse to healthcare providers or the police as mentioned below.

*Now one of the reasons is that the perpetrators themselves are the main financial supporter to these women. They have a thinking that if they disclose to us, we shall report to police and they [police] will go and arrest them. So, how will she survive? [Urban, Male, General Medical Doctor 10]*

*Like in this setting, I would say they [survivors] need the support in anyway. So, if they report, they know that I will be on my own. If my man beats me and then I go to report, they [perpetrators] will be imprisoned and then I will be on my own. So, there is that need of their [financial] support during this period. [Rural, Male, General Medical Doctor 4]*

#### Persistent safety concerns

Some healthcare providers, especially those in rural settings, revealed that some IPV survivors usually expressed concerns about continuous threats, including threats of death to themselves or their parents if perpetrators discover that they disclosed abuse to healthcare providers. It is commonplace for survivors to seek help from their immediate or closest family members, especially their parents; however, some respondents revealed that seeking help from family members may not necessarily make it a safe space for survivors as described in the excerpts below.

*They fear the husbands. If I talk about it, he is going to do it again. They are in fear all the time. Some have been threatened that “If you go to your parents’ home, I will find you there,” and we have seen women being killed from their parents, you divorce, you go to your parents’ place and then your husband follows you later [to their parent’s home] and kills you from there. [Rural, Male, ANC Nurse 1]*

*He batters you from his home, you reach time, and you say I cannot hold this anymore, you run to your parents if you are not from far, so they try by all means and get you from there. So you can imagine such a situation. So, they fear so much. If I go to my parents, I will get more problems, even to my parents. It will be now me and my parents. So, when we get those mothers, because there are some who deliver and say I am not going anywhere, because the moment I go back he is going to kill me. [Rural, Female, ANC Midwife 5]*

Respondents revealed that they have training needs that would help them provide or make appropriate referrals to stakeholders for supportive GBV care. This is highlighted in the quote below.

*You have to train us on how we can screen the mothers and where we can refer these mothers, and what can we do with these mothers. It may not be referring but having the skills that we can handle. So, if you can train us in how we can handle when a mother has such a problem, when gender-based violence is at this rate, you can handle like this and this one we cannot handle. [Rural, Female, ANC Midwife 7]*

### Distrust in healthcare providers

Our respondents also described survivors’ lack of trust in the confidentiality of IPV disclosed to healthcare providers. This is mentioned in the quotes below.

*I may think mothers are scared that we [healthcare providers] may involve the partners who may deny them custody and then they will not be able to know where to start from. So, they feel they are safe when they have not disclosed. [Urban, Female, ANC Midwife 13]*

*Since this is an African setting, these mothers at times fail to disclose because of fear of losing the marriage. They know that if I disclose, the ‘musawo’ [doctor] will call my husband, so sometimes they find it hard for them to disclose. [Urban, Female, ANC Midwife 9]*

### Normalizing Violence

#### Justifying Violence

Respondents mentioned that cultural beliefs condoning spousal abuse were held by some survivors. Recommendations to address these beliefs were suggested such as highlighting the need for societal-level sensitization to change such negative attitudes towards women as stated below.

Respondents mentioned that some cultural practices, such as dowry, contributes to the perception that women are ‘property’ and ‘owned’ by their male partner who perpetuates IPV.

*For this lady, he paid dowry and thinks he owns this person and you become his property. I think to this man he can do anything to the woman which is not fine. [Urban, Female, ANC Midwife 19]*

*This side, culturally they say a man is right to discipline his wife. For some men, they say that she is my wife and the pregnancy she is carrying is mine. So, I can do anything that I want to do with it. You know they are like this is my wife, and she is my property. Actually, they have a saying that ’namugula’ [I bought her], like you can go and buy your cow and then you say you will kill it. [Rural, Male, ANC Nurse 1]*

#### Abuse as an expression of affection

According to our respondents, some IPV survivors blamed themselves for abuse perpetrated against them by their partners while others were ambivalent about their views towards spousal abuse. For example, one provider mentioned that some survivors viewed abuse towards them as their partners’ way of expressing affection towards them, while other women justified abuse perpetrated against them by stating that partner abuse is culturally acceptable. This is mentioned in the excerpts below.

*Some do not talk about it because this is Africa. Someone thinks that if you don’t beat me that means you do not love me. Sometimes women blame themselves that I am the cause of the abuse. That is why women don’t talk about it, they take it as something normal to be abused. [Urban, Female, ANC Midwife 9]*

*Those cultures teach us that anything that a man does is normal. You are supposed to bear each and every torture because you are a woman, and he is a man. That is why most of the mothers do not talk. For example, if a man forces himself on you, when you are married, most of those mothers do not talk. How will you report when the culture tells us that what took us there is that [being abused]? [Urban, Female, ANC Midwife 13]*

*There is need to sensitize the community. They still have that saying that if a man does not beat you, then he does not love you. Some people keep quiet because that is the trend, so they need to sensitize communities. [Rural, Male, General Medical Doctor 4]*

### Unaware of human rights

Providers mentioned that many IPV survivors do not view IPV as a human rights violation, in part because of its normalization in several cultural communities which conflicts with the concepts of human rights. In addition, in some traditions in Uganda view the ‘ideal’ wife as one who does not disclose spousal abuse.

*They do not know gender-based violence hurts them. In fact, they don’t know about gender-based violence because according to culture, they teach them to be humble to their men. Even if a man does anything to you, you do not need to report a man to anyone, that is culture. If you go around talking about your husband then that means you are not a woman, in fact not a wife material, you are something else. So, they really cannot talk out because of culture and inferiority that is what really affects them. [Rural, Female, ANC Midwife 8]*

#### Self-blame and shame for abuse

Some respondents mentioned how a few women attending ANC clinics expressed feelings of self-blame or feeling ashamed for abuse perpetrated against them by their intimate partners. Some providers also mentioned that some women justify abuse. These sentiments are highlighted in the quotes below.

*Sometimes they blame themselves that I am the cause of the abuse. There is that saying … secrets for the family must remain in the family. They should not be taken to the outsiders. [Rural, Female, ANC Midwife 7]*

*Some women are really inferior to talk about, they really don’t talk about, I always call them inferiority, they really can’t talk out what happened, there are some mothers who really think if they talk out, they will be ashamed, so you will not know what happened to them. [Rural, Female, ANC Midwife 8]*

*Those cultures teach us that anything that a man does is normal, you are supposed to through each and every torture because you are a woman, and he is a man and that is why most of the mothers don’t talk. For example, if a man forces himself on you, when you are married, most of those mothers do not talk. How will you report when the culture tells us that what took us there is that.[Urban, Female, ANC Midwife 13]*.

## Discussion

This study explored healthcare worker experiences from provider-patient interactions with survivors attending antenatal care in Uganda. Our findings demonstrate an overall need to adopt survivor-centered approaches into healthcare service provision. Specific needs included addressing support services for survivors such as psychosocial, financial, medical and legal needs, raise awareness of existing GBV services and improve providers’ ability to offer GBV services or resources. These needs were perpetuated by survivors’ fear to disclose abuse due to potential consequences such as worsening or retaliatory partner abuse, fear or losing their marriage or partner’s financial support, concern for their own safety, stigma, and self-blame. In addition, IPV perpetrated against women seems ‘normal’ in some situations where survivors may justify IPV experienced by describing abuse as an expression of their partners affection towards the spouses. Our findings pointed out that IPV non-disclosure may persist since some survivors may have self-blame and instead feel ashamed suggesting that they may be unaware of their human rights.

Our investigation identified potential target areas at individual, facility, and societal level for interventions to address perceived survivor needs, and fears. For example, survivors who are unaware of existing GBV support services in their communities would benefit from GBV awareness raising campaigns that sensitize survivors on existing support services. On the other hand, survivors with anxiety to disclose abuse may feel powerless to change their situations due to several reasons highlighted in our study such as fear of losing their partners financial support, losing their marriage, retaliatory abuse, concerns of personal safety and life threats. Violence survivors who seek support services such as psychosocial or medical services should have their immediate concerns of safety addressed, particularly in cases when women fear retaliatory abuse from partners. Healthcare responses to survivor needs may be challenging. Prior research shows that facility-based clinicians, especially in rural settings, often do not routinely screen for IPV among healthcare seeking individuals [36]. Survivors in our study setting especially those attending rural ANC are already reluctant to report abuse because it violates societal norms that some traditions perceive as the ‘ideal’ wife, and/or because survivors fear that their partners will become more violent or retaliate financially. When healthcare providers do not screen women in primary healthcare settings, it may undermine efforts to sensitive survivors and communities as well about GBV and women’s rights. Our study showed that cultural norms that condone male perpetrated violence through societal ‘normalization’ of VAWG may worsen partner abuse.

According to healthcare providers, IPV survivors are unlikely to disclose abuse to anyone. The current study showed that women who suffer from IPV or rape, fear community stigmatization and tend to blame themselves for the abuse. Other women were afraid of divorce which could result in loss of partners’ financial support. Some survivors may not report abuse due to fears that it could make partners’ abuse worse or result in retaliatory violence. These fears appear to be well-founded, as providers reported that law enforcement and medical professions often reveal that women have disclosed abuse without providing protection from retaliatory abuse. Our study revealed that few GBV survivors are aware that spousal abuse is a violation of their human rights. In Uganda, where most cultures are patriarchal, it is challenging for female IPV survivors to freely express abuse suffered without the fear of retaliatory abuse from their intimate partners or community stigmatization. Interventions to change gender norms have shown promise in reducing negative attitudes of male partners perpetrating violence against their spouses [44]. Prior evidence revealed that survivors in Uganda are least likely to disclose IPV to healthcare workers, religious leaders, and police who are all key GBV response and prevention service providers. A complex interplay of individual and socio-environmental factors influences IPV disclosure among survivors such as when survivors perceive the negative outcomes of disclosure may outweigh the perceived benefits [45]. In fact, more women survivors of physical or sexual violence in Uganda sought help mostly from their own family, and their partner’s family with the least seeking help from doctors or medical personnel [13]. This could be due to traditional beliefs in several communities in Uganda where arbitration between couples’ families, and local council or tribal courts are culturally preferred. Abused women’s preferences of what they view as helpful provider responses to their disclosure of IPV included being treated respect and concern, protection, giving them control, responding immediately, providing survivors with options, providers’ being available later, and documenting their disclosure [46]. This prior study was in line with our findings that revealed persistent safety concerns and need to give survivors more control through financial empowerment and providing psycho-social support.

From a health systems perspective, non-disclosure of IPV has significant implications for clinical practice. It is important for healthcare providers to take low IPV disclosure into account when interacting with all patients who have sought healthcare services. Responding to women who have disclosed IPV involves an iterative process from rapport building, providing needed management / treatment, and making appropriate referrals to providers of GBV prevention and response services. Usually, the initial response to IPV reported to healthcare providers involves prioritizing management of the immediate medical emergencies and survivors’ safety related concerns. Non-disclosure of IPV is compounded by barriers to routine IPV screening by healthcare workers such as inadequate staffing levels, limited privacy, lack of provider IPV awareness and comprehensive GBV prevention and response training [35, 36]. Identifying survivors who express fears such as retaliatory abuse is important because of the life-threatening risk they are exposed to.

IPV survivors need financial empowerment or economic independence because of fear that their husbands who perpetrate abuse against them will not support them or their children. Previous studies show that financial empowerment through employment can mitigate the risk of IPV [47]. The lack of financial empowerment among survivors perpetuates coercive or abusive behaviour by male partners. Our study revealed that women experience different forms of partner coercion which includes financial, reproductive, emotional, and physical coercion. This diminishes the capacity of IPV survivors to achieve financial independence. Some survivors stay in abusive relationships as a coping mechanism due to fear of potential loss of financial support. A previous study conducted in rural Uganda showed that survivors who relied on their partners for financial support were less likely to report sexual gender-based violence than financially independent survivors [48]. Based on our findings, it is imperative that healthcare providers are well-prepared with the necessary resources to appropriately probe and identify IPV as well as collaborating with community-based stakeholders to address IPV. Such GBV response approaches include making appropriate referrals or linkage to psycho-social, legal/judicial, and financial support services.

Efforts aimed at addressing survivor’s economic needs such as income generating activities may have protective effects against IPV. This reinforces the rationale for integration of economic empowerment interventions into community-based, and/or health facility-based GBV prevention strategies. Financial/economic empowerment may lead to an increase in women’s income and wealth with a wider range of reproductive health benefits such as improving institutional delivery by skilled personnel [49], utilization of effective contraceptives [50], and optimal utility of antenatal care services [51]. Although empowerment may generally improve financial autonomy, this could also worsen IPV perpetration. Owning resources and wielding decision-making power regarding household expenditures challenges cultural norms and power dynamics of patriarchy. This may explain why several empowerment interventions aimed at reducing violence against women are multi-level i.e., individual, and structural [52]. Prior research demonstrated positive effects in changing negative norms and values that predispose to VAWG [53]. Survivors usually engage in arbitration through family members, traditional or community courts, religious leaders, and local council (LC) committees which are village level leadership teams elected from among community residents. However, the national policy on elimination of gender-based violence in Uganda [54] recommends that leadership at all administrative levels ensure immediate survivor safety and avoid evidence loss that could be used in cases of referred for medical assistance or law enforcement.

### Study implications

In order to adequately respond to survivor concerns and needs, it is important to reduce distrust of healthcare workers. Building trusting relationships between survivors and healthcare workers may contribute to improved IPV disclosure during patient-provider interaction and potentially increase rates of IPV detected and reported. Clinicians usually adopt patient-centered care approaches during patient-provider interaction that focus mostly on the patients presenting symptoms, and physical signs, as well as medical treatment. On the other hand, survivor-centered approaches have been widely applied in the context of war, and post- conflict settings [34, 55]. It is essential to ask survivors what they want or need in a culturally, and ethically responsive manner.

In cases of life-threatening abuse, GBV response strategies at health facility-level tend to be followed by referrals to institutions such as the police (CFPD), judicial system or involvement of community stakeholders such as arbitration councils or village courts involved in GBV prevention networks. Transformative initiatives to address IPV should be aimed at creating safe spaces around survivors to prevent further harm. Also, GBV sensitization strategies should include strategies to address spousal financial coercion. Therefore, we underscore the need for future studies to explore how awareness raising can be conducted in a culturally responsive manner that includes male partners. For example, future research could learn from experiences on how to address survivors’ distrust of providers and perpetrators during as partner notifications approaches in HIV care [56]. Partner notification is a Ministry of Health policy that encourages prioritizing couple ANC clinic attendance presents opportunities to leverage couple-dyads approaches in HIV prevention and care as partner notifications. From a programming perspective, the USAID’s strategy to GBV prevention and response encourages development and adaptation of best practices using novel screening and dissemination toolkits [57] to eliminate the different forms of violence [58].

Our study was limited by the fact that we elicited views of healthcare providers and not IPV survivors. The current study did not survey female survivors because it was not feasible at study implementation. However, it is worth noting that it is important to know what survivors’ needs and underlying demands are from their own perspective. Healthcare providers may not accurately express women’s own views of their needs [59]. Therefore, future research in these settings should explore the needs, challenges, and preferences directly from IPV survivors in Uganda.

## Conclusions

This study identified the perceived needs, and fears of IPV survivors from the perspective of rural and urban based, healthcare service providers in Uganda. We highlighted a potential opportunity for healthcare providers to create an environment that fosters IPV disclosure while ensuring that immediate and long-term survivor needs including safety concerns are addressed. Evidence generated from this study may inform GBV prevention and response strategies that incorporate survivor-centered approaches in Uganda.

## Data Availability

The datasets used and/or analysed during the current study are available from the corresponding author on reasonable request.
